# Modified-Release Pulmonary Delivery Systems for Labile Bioactives: Design, Development, and Applications

**DOI:** 10.3390/pharmaceutics17040470

**Published:** 2025-04-03

**Authors:** Shivani Nana, Mershen Govender, Yahya E. Choonara

**Affiliations:** 1 Wits Advanced Drug Delivery Platform Research Unit, Department of Pharmacy and Pharmacology, School of Therapeutic Sciences, Faculty of Health Sciences, University of the Witwatersrand, 7 York Road, Parktown, Johannesburg 2193, South Africa; 2 Wits Infectious Diseases and Oncology Research Institute, Faculty of Health Sciences, University of the Witwatersrand, Johannesburg 2050, South Africa

**Keywords:** respiratory system, physiological barriers, proteins and peptides, mRNA delivery, probiotics, nanosystems

## Abstract

Pulmonary delivery of bioactives has shown to be a promising route for the treatment of respiratory conditions, however, numerous physiological barriers, such as mucociliary clearance and immune responses, pose significant hurdles to treatment efficacy. These barriers specifically affect labile bioactives such as mRNA, peptides, proteins, and probiotics, which are susceptible to degradation due to the prevailing conditions. Various drug delivery platforms have been developed to address these challenges, including, among others, polymeric nanoparticles, micelles, liposomes, and solid lipid nanoparticles that encapsulate and protect the labile bioactives during formulation and administration, enabling improved bioavailability, sustained release, and enhanced formulation stability, while further modification of these platforms allows for targeted drug delivery. This review explores the advanced drug delivery systems that have been designed to protect and release labile active agents in a controlled and targeted manner to the lung, with a specific focus provided on the physiological barriers to effective pulmonary delivery and the formulation considerations to overcome these challenges. The outlook of this pertinent field of study has additionally been provided, highlighting the significant potential of the pulmonary delivery of labile bioactive agents for the prevention and treatment of a variety of respiratory ailments.

## 1. Introduction

The lungs represent a critical interface for therapeutic interventions such as asthma, chronic obstructive pulmonary disease (COPD), and pulmonary cancers, with their extensive surface area, rich blood supply, and thin epithelial barrier, facilitating the absorption of therapeutic agents for both local and systematic effects [1]. Targeted delivery of drugs (or other bioactives) to lung tissue additionally enhances the drug therapeutic index for lower doses, thereby reducing adverse effects. Furthermore, by using mechanisms to control the drug release, therapeutic concentrations of the drug can be maintained over extended periods to improve patient compliance for better treatment outcomes. To achieve this fate, drug delivery to the lungs requires various biological barriers to be traversed, with the mucosal layers and alveolar structures being significant barriers to optimal drug delivery via the oral or intravenous routes [2]. Oral drug delivery intended to reach the lung tissue furthermore often results in premature degradation in the GIT and/or subjected to first-pass metabolism in the liver, while intravenous administration fails to provide site-specific drug action. Moreover, the physicochemical properties of drugs can lead to rapid clearance or degradation before exerting a therapeutic effect [3].

**Figure 1:**
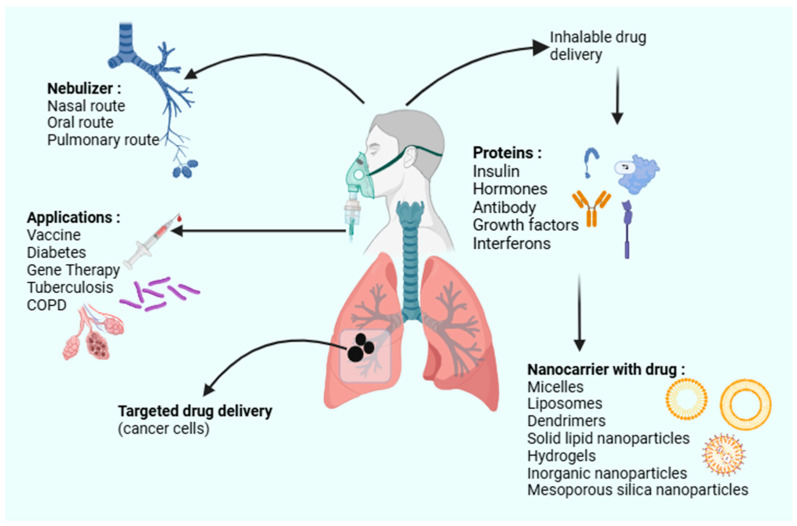
A schematic of the various inhalable drug delivery systems utilizing nanocarriers (created with BioRender.com).

Site-specific therapeutic interventions for lung diseases often include labile bioactives, such as peptides, proteins, and nucleic acids that target specific cellular pathways. In addition to the physiological barriers, the instability of these bioactives during formulation and administration presents a critical challenge for effective pulmonary delivery [4]. This necessitates the development of advanced drug delivery systems that can provide targeted and controlled bioactive release while maintaining the pharmaceutical stability of the labile bioactives, as depicted in Figure 1. Advancements in drug delivery, such as the use of nanoparticles, liposomes, and hydrogels, offer promising solutions to enhance the delivery of labile bioactives to the lung, facilitate targeted delivery, and control release profiles to meet the specific therapeutic function of various peptides, proteins, nucleic acids, vaccine candidates, and probiotics [5,6]. Peptides and proteins in particular have limited stability and permeability across membranes, while nucleic acid-based bioactives, such as mRNA, require protection against enzymatic breakdown and cold storage to maintain an effective immunogenic response [7,8,9,10]. Similarly, probiotics comprise live bacteria that need to remain viable for targeted lung microbiome interventions [11].

This review therefore aims to provide a concise incursion of the current landscape of advanced delivery systems for the targeted and controlled release of labile bioactives to the lung for local and systemic effects. A specific focus on the key barriers to effective pulmonary delivery is provided with the assimilation of various delivery mechanisms designed to overcome these challenges. In addition, the evolving trends in this field are outlined for research in this domain to be advanced and disrupted [12,13,14,15,16,17].

## 2. Physiological Barriers to Effective Pulmonary Delivery of Bioactives

**Figure 2:**
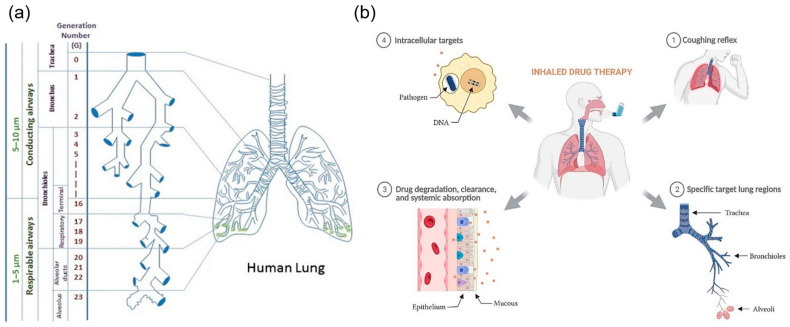
A graphical representation of (**a**) the branched airway network of the lungs illustrating the deposition pathways, the various lung regions involved, and (**b**) the key biological barriers that affect drug absorption, including epithelial barriers, mucociliary clearance, and immune responses in a healthy patient (adapted with permission from Kunda et al. [20], © 2012 Springer Science Business Media and Plaunt et al. [24], © 2022 by the authors. Licensee MDPI, Basel, Switzerland).

The respiratory system consists of a hierarchical network of structures optimized for gaseous exchange. Of these structures, the alveoli represent the primary site for drug absorption due to its extensive surface area (estimated at 50–100 m^2^), proximity to pulmonary capillaries, and a thin alveolar-capillary membrane (~0.2–0.6 µm) [18,19]. The respiratory system, however, constitutes defense mechanisms that protect the body against foreign and potentially toxic substances. These barriers (i.e., physical, chemical, biological, and immunological), as summarized in Table 1, present unique challenges against effective pulmonary delivery. It is therefore crucial to assess the physicochemical properties of the bioactives intended for lung delivery to facilitate overcoming these barriers and affect therapeutic responses. Figure 2 additionally provides a graphical representation of the branched airway network of the lungs and the key biological barriers that affect drug absorption, including epithelial barriers, mucociliary clearance, and immune responses in a healthy patient.

**Table 1:** Physicochemical and physiological barriers to effective pulmonary drug delivery.

Barrier Type	Barrier	Description	References
Physical	Branching airway network	Complex airway geometry can hinder the passage of inhaled particles, especially deeper in the lungs.	[20,21,22,23]
	Particle size and aerodynamics	Optimal particle size for deep lung delivery is 1–5 µm; larger particles deposit in upper airways; smaller ones may be exhaled.	
	Mucociliary clearance	Airways are lined with cilia and mucus, which trap and remove foreign particles, reducing drug retention.	
Chemical	Chemical composition of lung fluids	Lung fluids contain various ions and surfactants that can alter drug solubility and stability.	[24,25,26]
	Lung fluids contain various ions and surfactants that can alter drug solubility and stability	Enzymes (e.g., proteases, esterases, cytochrome P450) degrade bioactive molecules, reducing drug stability. The presence of lung-specific enzymes further breaks down bioactives before absorption, reducing their effectiveness.	
	pH variation	pH varies from 5.5 to 7.6 along the respiratory tract, affecting drug solubility and stability.	
Biological	Epithelial barrier	Epithelial cells form a stringent barrier that regulates substance passage, requiring drugs to navigate effectively.	[27,28,29]
	Lung diseases (e.g., cystic fibrosis, COPD)	Chronic diseases lead to excess mucus, inflammation, and bacterial biofilms that hinder drug penetration.	
	Microbiome and bacterial biofilms	Dense bacterial biofilms act as barriers, preventing drug penetration and reducing therapeutic efficacy.	
	Allergic reactions	Allergic reactions can lead to airway constriction, reducing drug delivery efficiency.	
Immunological	Macrophage clearance	Macrophages recognize and clear inhaled particles, including therapeutic drugs, reducing their bioavailability.	[30,31]
	Inflammatory responses	Immune-mediated inflammation can lead to lung tissue damage and further hinder drug absorption and retention.	
	Immune responses	Macrophages and lymphocytes neutralize foreign substances, impacting drug effectiveness.	

## 3. Advanced Delivery Systems for Pulmonary Delivery of Labile Bioactives

**Figure 3:**
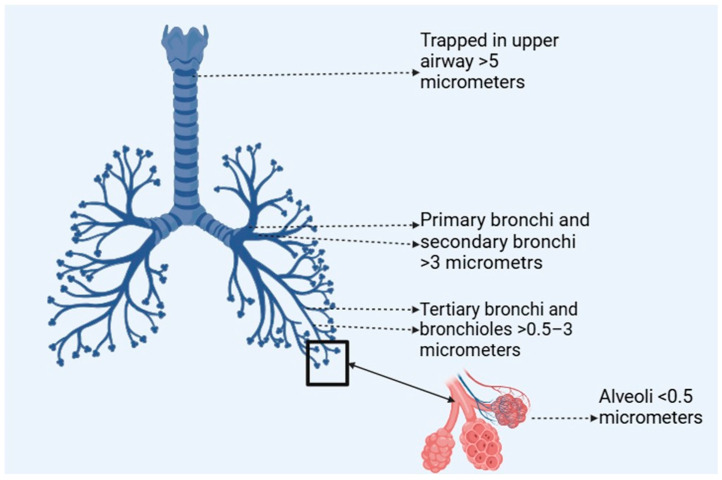
Particle size-dependent lung deposition (created with BioRender.com).

Advanced drug delivery systems utilizing, specifically, micro- and nanoparticles offer promising solutions to overcome the inherent challenges of pulmonary drug delivery, particularly for labile bioactives. The design of these platforms focuses on enhancing drug localization, targeted delivery, and controlled release while addressing the mechanical and physiological barriers within the lungs [24]. Figure 3 displays the influence of particle size (micro-sized particles (1 to 5 µm) and nano-sized particles (typically 100 to 1000 nm)) to bypass the upper respiratory tract and deposit directly into deeper lung regions, such as the alveoli, crucial for efficient drug accumulation or absorption. This precision ensures that drugs are delivered where they are most needed, improving therapeutic efficacy and reducing systemic side effects.

The protection of labile bioactives from the harsh enzymatic environment of the respiratory tract primarily involves the encapsulation of sensitive molecules within particulate systems, providing both stability and resistance to enzymatic degradation [32]. Encapsulation also facilitates controlled drug release over extended periods, maintaining therapeutic levels in the lung and reducing dosing frequency, which is essential for enhancing patient compliance. Surface modifications of these nanoparticles, such as with polyethylene glycol (PEG; PEGylation) or the addition of specific ligands, further enhance their ability to penetrate mucus and increase cellular uptake by lung cells [33]. This is often achieved by optimizing the particles’ surface properties to improve their interaction with the lung’s biological media, thereby enhancing penetration and bioavailability.

The clinical implications of these particulate systems in respiratory therapies are profound as ongoing research continues to focus on enhancing their targeting capabilities, reducing potential toxicities, and optimizing manufacturing processes for wider applications [34]. Numerous studies have demonstrated the effectiveness of particulate systems in protecting labile bioactives and improving delivery to the lungs; for example, liposomal formulations encapsulating antibiotics like amikacin have shown enhanced stability and reduced enzymatic degradation in the respiratory tract, enabling sustained drug release and improved efficacy against pulmonary infections such as *Pseudomonas aeruginosa* in cystic fibrosis patients [35].

**Figure 4:**
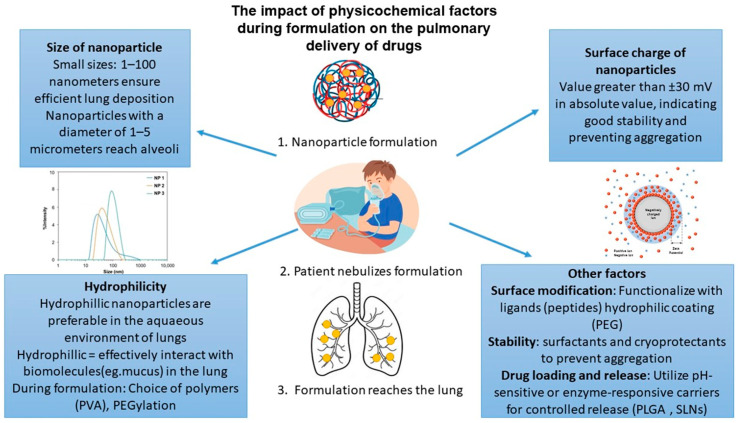
Physicochemical factors influencing pulmonary drug delivery.

Similarly, polymeric nanoparticles loaded with peptides have been investigated, where encapsulation significantly increased peptide stability and bioavailability, ensuring therapeutic levels in the lungs over extended periods with reduced dosing frequency [36]. Specifically, in a study focused on PEGylated lipid nanoparticles for mRNA delivery, this platform demonstrated superior delivery to lung epithelial cells with reduced inflammatory responses, a critical step in advancing therapies for genetic disorders [37]. Additionally, inhalable chitosan-based nanoparticles loaded with siRNA have been shown to silence inflammatory mediators effectively, illustrating their potential in treating COPD and asthma [38]. The above two studies highlight the importance of evaluating physicochemical properties during nanoparticle formulation for enhanced pulmonary delivery (Figure 4 provides a summary of the physicochemical factors influencing pulmonary drug delivery). The following sections provide for the advanced drug delivery systems that have been developed for the delivery of labile bioactive to the lungs, characterized based on their loaded bioactives. These advanced pulmonary delivery systems are generally biocompatible, well tolerated, and, in some cases, FDA-approved. While most exhibit low toxicity and minimal immunogenicity, some may cause local inflammation, lipid accumulation, or dose-dependent effects. Careful formulation and evaluation are therefore essential to ensure their safety for clinical use (a summary of the characteristics and applications of these drug delivery systems has been provided in Table 2).

### 3.1. Protective Strategies Against Degradation of Proteins and Peptides for Pulmonary Delivery

Typical examples of proteins that have been delivered to the lungs include insulin, interferon-alpha (IFNα), human growth hormone (hGH), recombinant human deoxyribonuclease (rhDNase), and calcitonin [15]. To ensure the effective pulmonary delivery of labile bioactives, various formulation strategies have been developed to overcome enzymatic degradation, poor stability, and rapid clearance. Insulin, for instance, is encapsulated in protective carriers such as nanoparticles or liposomes to enhance its stability and absorption while minimizing degradation and is delivered through the pulmonary route to achieve systemic effects, providing a non-invasive alternative to injections [59], while IFNα, commonly used for viral infections and cancer, benefits from PEGylation or lipid-based formulations that protect it from enzymatic breakdown and improve retention in the lungs and thus benefits from pulmonary delivery by directly targeting respiratory infections and enhancing therapeutic efficacy [60]. Similarly, human growth hormone (hGH), which is stabilized using polymeric carriers to prevent degradation before reaching systemic circulation, demonstrates effective absorption through the lungs’ large surface area, and rhDNase, used for cystic fibrosis treatment, is often formulated in aerosolized solutions with stabilizing excipients to maintain bioactivity during inhalation and deposition in the lung, aiding in breaking down mucus in the lungs, thus improving respiratory function [61]. Calcitonin, a peptide hormone for osteoporosis, has additionally been encapsulated in biodegradable microparticles or modified with absorption enhancers to improve pulmonary uptake [62,63].

Across these formulations, strategies such as encapsulation, PEGylation, surfactant stabilization, and mucoadhesive materials play a crucial role in overcoming degradation challenges, ensuring prolonged drug retention, controlled release, and enhanced therapeutic efficacy. These proteins further benefit from pulmonary delivery due to the lungs’ extensive absorptive surface area, high vascularization, and rapid onset of action [64]. The following sections provide an in-depth review of the platforms that have been developed for the pulmonary delivery of proteins and peptides.

#### 3.1.1. Spray-Dried Inhalable Powders

Spray-dried inhalable powder formulations extensively cover the development of dry powder inhalers (DPIs) for the pulmonary delivery of protein and peptide drugs, highlighting several critical aspects. The stability of these polymers emphasizes that maintaining polymer stability during spray-drying is crucial, particularly by employing stabilizing excipients like sugars and polyols (e.g., mannitol and trehalose) that preserve the structural and biological integrity of the loaded molecules [65].

The delivery of proteins and peptides through DPIs presents a promising non-invasive alternative to injections, providing targeted drug delivery directly to the lungs and enhanced stability in their solid form. This was specifically noted in a study by Depreter and Amighi, who detailed the preparation and evaluation of DPIs for insulin delivery [64]. In their study, Depreter and Amighi developed dry powder inhaler (DPI) formulations for insulin delivery using high-pressure homogenization followed by spray-drying. To enhance the stability and dispersibility of the insulin particles, they applied a lipid coating composed of a mixture of cholesterol and phospholipids. This lipid coating served to reduce residual moisture content and improve the aerodynamic properties of the particles, facilitating deeper lung deposition. The use of these physiological lipids aligns with their role in stabilizing the protein and enhancing dispersibility. The study concluded that adjustments in these parameters significantly impacted the physicochemical properties and aerodynamic performance of the powders, with lower inlet temperatures leading to less efficient drying and inconsistent particle size, while higher temperatures caused partial denaturation of insulin, reducing its bioactivity. The uniformity of the delivered dose, assessed using a ‘Uniformity of the Delivered Dose’ device, showed that all capsules delivered between 94.5% and 102.5% of the mean delivered dose, ensuring consistent dosing. In terms of lung deposition, aerodynamic evaluation using a Multi-Stage Liquid Impinger revealed that the fine particle fraction of the formulations ranged from 46% to 63%, compared to only 11% for raw insulin powder, with the mass median aerodynamic diameter of the optimized formulations between 2.7 µm and 3.5 µm, demonstrating their suitability for deep lung deposition, which is critical for systemic absorption and therapeutic efficacy, thus overcoming pulmonary barriers. These results underscore the potential of DPI formulations for effective and non-invasive insulin delivery, with the lipid-coated formulations exhibiting excellent aerodynamic features and stability, supporting their potential for effective pulmonary delivery [64].

The feasibility of delivering therapeutic antibodies for asthma treatment via inhalation has additionally been evaluated in a study by Pan et al. [66]. This study utilized spray-drying and spray-freeze-drying methods to formulate dry powders of an anti-IL-4 receptor monoclonal antibody (mAb), with 2-hydroxypropyl-beta-cyclodextrin (2HPβCD) included as a stabilizing excipient. The powders were characterized for their aerodynamic properties and protein stability, showing emitted fractions exceeding 80% and fine particle fractions around 50%, suitable for deep lung deposition. The results of this study determined that the mAb retained antigen-binding ability and bioactivity after processing, with satisfactory stability over a year of storage at ambient conditions. These findings also demonstrated the potential of inhalable dry powder formulations for non-invasive pulmonary delivery of biologics, emphasizing the importance of maintaining protein stability and achieving effective aerosol performance for therapeutic applications [66].

In another study by Amaro et al., sugar-based salmon calcitonin-loaded nanoporous/nanoparticulate microparticles (NPMPs) were produced using a mini-spray-dryer, possessing aerodynamic and physicochemical properties that make them highly suitable for pulmonary delivery through DPIs. The formulation incorporated trehalose and raffinose as stabilizing agents to protect the protein during spray-drying and ensure structural integrity. Hydroxypropyl-β-cyclodextrin (HPβCD) was included to enhance solubility and stability, while leucine was added to improve aerosolization and prevent particle aggregation. Additionally, polyvinyl alcohol (PVA) was used to aid in particle stabilization and film formation. These excipients played a crucial role in ensuring the final formulation’s suitability for inhalation, aligning with their functions in enhancing bioavailability and stability [67]. Aerodynamically, the microparticles achieved fine particle fractions of 45–86% for particles with diameters under 5 µm and mass median aerodynamic diameters ranging from 1.9 to 4.7 µm, enabling effective deposition in the deep lung regions. The powders exhibited good dispersibility, ensuring efficient delivery to the alveolar space. Physicochemically, the particles were spherical, porous, and small (approximately 2 µm in size), with an increased specific surface area enhancing their flowability and deposition efficiency. The powders were amorphous, contributing to the stability of the encapsulated bioactive molecules, and the raffinose-based formulations retained nearly 100% of their bioactivity, demonstrating superior stability compared to trehalose-based systems. These properties collectively highlight the potential of the NPMPs as an effective, stable, and non-invasive delivery system for therapeutic peptides like salmon calcitonin.

Through the various research studies that have investigated, spray-dried powder has been shown to be effective for numerous bioactives, with the advantages of using DPI formulations, including non-invasive delivery and ease of administration with increased product stability and efficacy. These advantages are specifically useful in the delivery of labile bioactives, where enhanced protection and stability are essential for predictable therapeutic responses.

#### 3.1.2. Liposomal Encapsulation

Liposomes have been used as drug delivery systems for the targeted delivery of proteins and peptides to the lungs. These spherical vesicles comprise phospholipids, a type of polymer that forms bilayers, encapsulating therapeutic agents within their aqueous core. This encapsulation enhances the drugs’ stability and facilitates their efficient delivery to lung tissue. The stability of these liposomal systems is critical for maintaining the therapeutic efficacy of the bioactive, which is achieved through the incorporation of cholesterol into the liposomal membrane. This forms a lipid bilayer barrier, thus protecting the bioactive against enzymatic degradation. Lipid-based composition mimics natural cell membranes, facilitating fusion with lung epithelial cells for enhanced drug absorption.

In the studies by Huang and Wang [56], Abu-Dahab et al. [57], and Woodle et al. [58], the applications of liposomal formulations for the pulmonary and systemic delivery of labile therapeutic agents were explored, highlighting their shared benefits and adaptations for different medical needs. The studies share several methodological similarities in the preparation and optimization of liposomes. Huang and Wang utilized membrane destabilizing and detergent dialyzing techniques to encapsulate insulin with high efficiency and generate liposomal aerosols suitable for pulmonary delivery, while Abu-Dahab et al. employed solvent evaporation to prepare liposomes, integrating cholesterol to enhance stability during nebulization. Woodle et al. additionally employed PEGylation to extend circulation times and functionalized liposomes with targeting ligands for site-specific delivery. Nebulization emerged as a common method for delivering liposomal formulations in these studies, with the appropriate particle sizes (~1 μm) ensured for deposition in the lungs, reducing clearance by coughing or mucociliary transport. Functionalization with additives like cholesterol and lecithin (Abu-Dahab et al.) and PEG (Woodle et al.) further enhanced liposomal stability, bioadhesion, and targeted interactions, improving their potential for effective pulmonary delivery [57,58].

Specifically, within these studies, Huang and Wang reported that the insulin-encapsulated liposomes delivered via ultrasonic nebulization significantly reduced plasma glucose levels in diabetic mice by up to 200 mg/dL compared to controls with results demonstrating sustained glucose reduction over several hours, confirming effective pulmonary delivery and prolonged drug action. Abu-Dahab et al. additionally demonstrated that the lectin-functionalized liposomes achieved a 30–40% increase in cellular binding to human alveolar epithelial cells (A549 cell line) compared to non-functionalized liposomes, even in the presence of lung surfactant. The stability of these liposomes during nebulization was notable, with the cholesterol-containing formulations exhibiting only 8% leakage of encapsulated hydrophilic markers, compared to 15–20% leakage in cholesterol-free liposomes, while Woodle et al. displayed that the PEGylated liposomes prolonged systemic delivery of peptides like vasopressin, with biological activity sustained for up to one month compared to less than one week with free vasopressin. Additionally, in a separate analysis, Woodle et al. investigated the sustained delivery of vasopressin using sterically stabilized liposomes (SSL), which extended the peptide’s biological activity significantly compared to free vasopressin, highlighting the versatility of liposomal delivery systems in improving therapeutic profiles while reducing systemic side effects and enhancing retention at the target site.

The delivery of drugs via liposomes to the lungs can therefore be accomplished through nebulization, where the liposomal suspension is transformed into an aerosol that patients can inhale. Aerosolized liposomes are small (20–1000 nm) and uniformly dispersed, allowing them to penetrate deeper lung regions (bronchioles and alveoli) rather than being trapped in the upper respiratory tract and cleared by mucociliary action. This method ensures targeted drug delivery directly to the lung tissue and minimizes systemic side effects. The studies reviewed share several key similarities in their approach to utilizing liposomes as drug carriers, highlighting the versatility and efficacy of these systems, with the liposomes further demonstrating the ability to enhance the stability of sensitive therapeutic agents by providing a protective environment that prevents degradation during delivery processes. Targeted delivery was another consistent theme, with liposomal formulations achieving improved retention at the desired site, whether for pulmonary therapies or tumor sites for potential cancer immunotherapy, thereby enhancing therapeutic outcomes and minimizing systemic side effects. These shared features illustrate the adaptability of liposomal systems to address a wide range of drug delivery challenges, emphasizing their role as a robust platform in modern therapeutics.

#### 3.1.3. Polymeric Nanoparticles

In pulmonary drug delivery, polymeric nanoparticles have emerged as a vector for the targeted delivery of pharmaceutical agents directly to the lungs. Among these, nanoparticles containing PLGA stand out due to their FDA-approved status, biocompatibility, and biodegradability, which facilitate sustained release kinetics [68]. Chitosan, a natural polysaccharide, is frequently employed for its mucoadhesive properties, which enhance drug retention in the lungs and improve absorption. Similarly, alginate is often used in combination with chitosan to form hydrogel-based nanoparticles, providing a protective matrix for sensitive bioactives. Poly(lactic acid) (PLA), a structurally related polymer to PLGA, degrades more slowly, making it suitable for extended drug release applications. Additionally, poly(alkyl cyanoacrylates) are synthetic polymers that enable the formulation of biodegradable nanoparticles capable of encapsulating both hydrophilic and hydrophobic drugs, further enhancing pulmonary drug delivery. The selection of these polymers depends on the specific properties of the drug being delivered, the required release profile, and the overall formulation strategy to maximize therapeutic efficacy. The recent literature has demonstrated the utility of PLGA nanoparticles in pulmonary delivery applications, focusing particularly on proteins and peptides [69]. These nanoparticles not only prevent the degradation of these molecules but also enhance the bioavailability and therapeutic efficacy of the delivered agents. Stability studies have further shown that PLGA nanoparticles maintain their integrity and functionality in simulated lung fluids and against physical stresses typical of nebulization processes, showcasing their suitability for inhalational therapies [69].

Preclinical studies in animal models have been crucial in clarifying the pharmacokinetics and pharmacodynamics of PLGA-based nanoparticle systems. Notably, studies involving rat models have demonstrated that these nanoparticles can effectively deliver and release proteins such as erythropoietin within the lung tissue over extended periods [39,40]. Specifically, release can persist for up to 10 days for erythropoietin encapsulated in PLGA nanoparticles, and the distribution of these nanoparticles is uniform and sustained throughout the lung. The nanoparticles used in these studies are typically around 160 nm in size, which is within the optimal range for deposition in the alveolar region without being cleared by alveolar macrophages. These findings were further validated by in vitro studies that show high cytocompatibility and minimal cytotoxicity of PLGA nanoparticles, even at higher concentrations, ensuring safety and efficacy [41,42,43].

#### 3.1.4. Conjugated Proteins

**Figure 5:**
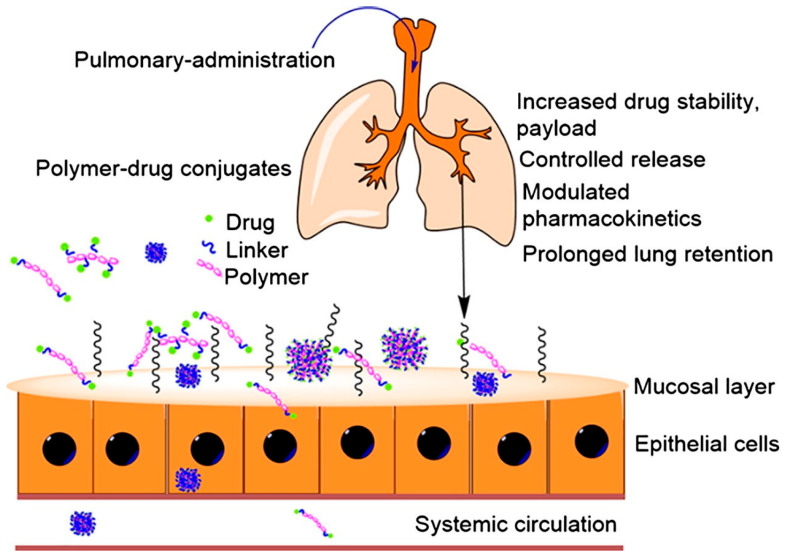
Schematic representation of pulmonary administration using polymer–drug conjugates (reproduced with permission from Marasini et al. [70], © 2017 Elsevier Ltd., London, UK).

Polymer–drug conjugates are platforms that traverse the mucosal layer to reach epithelial cells, facilitating targeted delivery and effective therapeutic outcomes in the lungs, as depicted in Figure 5. The utilization of polymer–drug conjugates as inhalable drug delivery systems is of particular focus in the delivery of therapeutic proteins and peptides, with the employment of polymers such as PEG, PLGA, chitosan, PVA, poly(ethylene oxide) (PEO), poly(caprolactone) (PCL), and hyaluronic acid instrumental in modifying the pharmacokinetic profiles and biodistribution of therapeutic agents administered via the pulmonary route [70]. The advantages of such delivery systems include enhanced drug absorption due to the high vascularization and large surface area of the lungs and prolonged retention times that are beneficial for treating lung-resident diseases. Empirical evidence from animal studies provides substantial support for the efficacy of these delivery systems. For example, modified polymers like PEG have demonstrated the ability to improve the penetration and retention of chemotherapeutic agents, facilitating sustained therapeutic effects [71]. The stability of these conjugates is, however, crucial for maintaining the bioactivity of therapeutic compounds during and after pulmonary delivery and is a critical consideration in the design of polymer–drug conjugates for labile bioactives [72].

One such study that focused on the development of a polymer–drug conjugate was by McLeod et al., who evaluated the use of PEGylation to enhance lung and systemic exposure of IFNα2 following intratracheal administration in rats [73]. By attaching PEG chains to IFNα2, the researchers aimed to improve its stability and retention in the lungs. The study compared different PEG chain lengths (12 kDa and 40 kDa) and found that the 31 kDa PEGylated construct significantly increased lung retention and decreased systemic exposure compared to the 60 kDa construct. Specifically, the 31 kDa PEGylated IFNα2 increased lung exposure by 50% and systemic exposure by 100% compared to the native protein. The study therefore concluded that optimal PEGylation can enhance the delivery and efficacy of inhaled protein therapeutics for treating lung diseases while minimizing systemic side effects [73].

In an interesting area of research, clinical trials have also begun to demonstrate the potential of these systems in human applications, suggesting promising strategies for managing respiratory diseases with reduced systemic side effects. These findings demonstrate the potential of inhalable polymer–drug conjugates for the treatment of pulmonary diseases, providing a compelling case for further clinical development and investigation into these drug delivery platforms [74,75].

#### 3.1.5. Solid Lipid Nanoparticles

Solid lipid nanoparticles (SLNs) have received significant attention as a drug delivery system as they enhance the therapeutic agents’ bioavailability while potentially protecting the loaded bioactive against degradation. Using biocompatible and biodegradable lipids, SLNs offer a promising alternative to traditional colloidal carriers such as emulsions, liposomes, and polymeric nanoparticles [76]. SLNs are typically formulated using physiological lipids and are further stabilized using surfactants such as lecithin and polysorbates [44].

**Figure 6:**
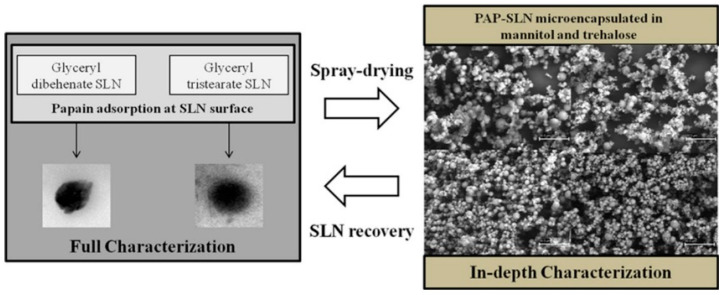
SLNs developed for the delivery of PAP to the lung, scale bar: 30 μm (reproduced with permission from Gaspar et al. [45], © 2016 Elsevier B.V, Amsterdam, Netherlands).

Ensuring the stability of drugs within SLNs is a critical factor, especially with labile active drug delivery. Encapsulation within the lipid matrix of SLNs protects the drug from degradation caused by environmental factors such as pH, temperature, and enzymatic activity. This was confirmed in a study by Gaspar et al. [45], where the stability potential of SLNs containing the protein papain (PAP) was assessed using SDS-PAGE analysis. The results of the study showed that the structural integrity of PAP was preserved during the adsorption process and after release from both the SLNs and the spray-dried powders. This indicated that the protein did not undergo significant degradation during the microencapsulation and spray-drying processes used for pulmonary delivery. The SLNs were prepared form glyceryl dibehenate and glyceryl tristearate through hot high-shear homogenization, followed by the adsorption of PAP. These PAP-SLN formulations were then spray-dried with mannitol and trehalose to create dry powders suitable for inhalation. The resulting powders, as depicted in Figure 6, exhibited aerodynamic diameters of approximately 5–6 µm, ideal for deep lung deposition, specifically targeting the alveolar region for enhanced therapeutic effect. Additionally, the powders demonstrated real densities ranging from 1.43 to 1.81 g/cm^3^, contributing to their favorable aerodynamic behavior. Additionally, PAP is released from the dry powders to a greater extent than non-spray-dried SLNs, suggesting effective release mechanisms in the designed formulations while preserving protein stability [45]. The improved pharmacokinetic profile and enhanced bioavailability of PAP encapsulated in SLNs therefore highlight the potential of this delivery system for clinical applications.

#### 3.1.6. Micelles

Polymeric micelles have also become an essential nanotechnology-based pulmonary drug delivery system due to their ability to improve the solubility, stability, and bioavailability of poorly water-soluble drugs. This makes micelles particularly useful for delivering labile active drugs [46]. Polymeric micelles are typically formed from amphiphilic block copolymers, which self-assemble in aqueous environments, with common polymers including PEG, PCL, and polylactic acid. These polymers can be tailored to control the size, drug loading capacity, and release profile of the micelles [47].

Micelles protect encapsulated drugs from degradation by isolating them within the hydrophobic core. This protection extends the drug’s shelf-life and maintains its therapeutic efficacy. For instance, encapsulating curcumin in micelles has been shown to protect it from degradation and enhance its stability in biological environments [45]. Micelles can also deliver drugs through various mechanisms, including passive targeting, active targeting, and triggered release. Passive targeting exploits the enhanced permeability and retention (EPR) effect, allowing micelles to accumulate in tumor tissues. Active targeting involves modifying the micelle surface with ligands that bind to specific receptors on target cells, enhancing drug delivery to specific tissues. Triggered release mechanisms, such as pH-sensitive or redox-sensitive micelles, can further release the drug in response to specific environmental stimuli within the body [46].

In a study evaluating the efficacy of micelles and liposomes as carriers for delivering proteins to tumors in a mouse model of subcutaneous Lewis lung carcinoma, these carriers were formulated using PEG, which enhanced their stability, biocompatibility, and circulation time in the bloodstream [48]. The researchers prepared long-circulating formulations by incorporating PEG into the micelles and liposomes, reducing their clearance by the immune system and allowing for prolonged systemic presence. The encapsulated proteins demonstrated efficient loading, with a retention efficiency exceeding 85%, and exhibited significant accumulation in tumor tissues, with protein concentrations in the tumor increasing by over 200% compared to free protein administration. The targeted delivery enabled by these carriers facilitated sustained release and greater therapeutic availability at the tumor site, leading to a pronounced reduction in tumor growth in the treated mice. Additionally, the study observed minimal systemic toxicity, highlighting the safety profile of these polymer-based carriers. These findings showcase the potential of the PEGylated micelles and liposomes as delivery platforms for therapeutic proteins, offering enhanced targeting and improved treatment outcomes for lung cancer and other solid tumors [48].

In another study focusing on the delivery of salmon calcitonin, the objective was to enhance the drug’s stability against lung enzymes and improve its transepithelial absorption. The research utilized in vivo experiments conducted in rats, where salmon calcitonin was delivered through aerosolized intratracheal administration. The results demonstrated that micelles significantly increased the drug’s bioavailability by 160 ± 55%, which is over 60% higher compared to the free drug solution [43]. In another study aimed at delivering a labile bioactive to the lung, polymeric micelles were used to encapsulate citraconic-modified ovalbumin [49]. The in vivo experiments revealed that the micelles elicited a strong immune response, as evidenced by significantly increased levels of specific IgG, IgG1, and IgG2a in mice seven days after the third immunization, compared to the free antigen. The PEG-lipid-based micellar complexes developed significantly enhanced the bioavailability of the salmon calcitonin compared to the free drug solution. Specifically, the relative bioavailability of salmon calcitonin delivered via micelles was increased by over 60%, with micelles achieving a mean bioavailability of 160% ± 55% when compared to the free antigen. Additionally, the micellar formulation provided a sustained and higher plasma concentration, significantly improving drug pharmacokinetics (AUC_inf_ = 23 ± 8 for the micelles compared to 14 ± 6 min·ng/mL/μg·kg for the free drug solution). These findings underline the potential of micellar systems to enhance the stability, absorption, and efficacy of protein-based drugs delivered to the lungs.

### 3.2. Advanced Nucleic Acid Protection and Delivery

The ability of the nucleic acids mRNA to encode virtually any protein makes it a versatile tool for various therapeutic applications, including cancer treatment and immunoregulation [77,78]. The delivery of mRNA relies heavily on its encapsulation within systems that can protect it from degradation and facilitate cellular uptake [79]. Clinical evidence supporting the use of mRNA-based therapies is emerging, particularly following the success of mRNA vaccines for COVID-19 [80,81,82]. The following sections provide an in-depth review of the platforms that have been developed for the pulmonary delivery of nucleic acids.

#### 3.2.1. Lipid Nanoparticles

**Figure 7:**
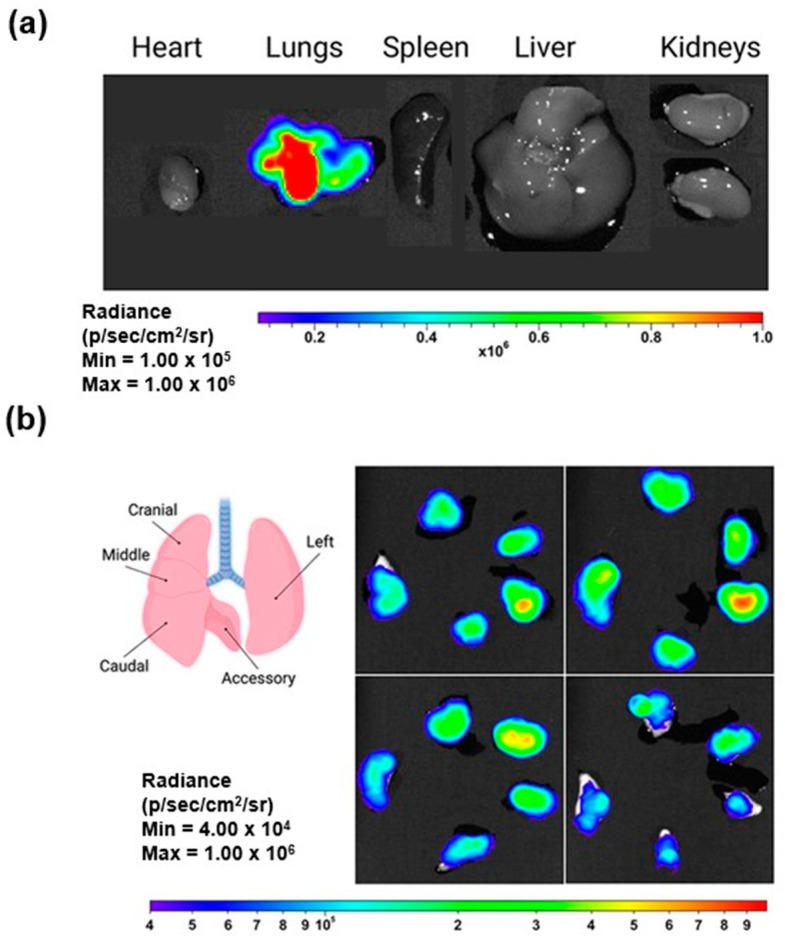
Images of LNP inhalation resulting in selective transfection within the lungs: (**a**) representative bioluminescent images of isolated organs demonstrate localized luciferase expression specifically in the lungs, and (**b**) LNP inhalation results in selective transfection within the lungs (reproduced with permission from Kim et al. [83], © 2022 American Chemical Society, Washington, DC, USA).

In a study conducted by Kim et al. [83], LNPs for delivering mRNA to the lungs via inhalation were developed, focusing on optimizing intracellular delivery, with luciferase mRNA used as a reporter to assess transfection efficiency. The LNP formulation was optimized with β-sitosterol and PEG lipids, enhancing stability during nebulization and mucus penetration. The optimized LNPs further demonstrated efficient mRNA delivery and localized protein expression in the lungs, highlighting their potential for targeted pulmonary gene therapies with minimal off-target effects. Results from the study revealed that these LNPs retained over 90% mRNA encapsulation efficiency and maintained particle sizes of 100–130 nm post-nebulization. After nebulization, the LNPs exhibited significantly higher luciferase expression in mouse lung tissue, with up to a 12-fold increase compared to standard LNP formulations. Moreover, repeated administration demonstrated sustained protein production in the lungs, with no significant pulmonary or systemic toxicity observed. When tested in cystic fibrosis transmembrane conductance regulator (CFTR)-deficient mice, the inhaled LNPs successfully delivered the therapeutic mRNA, leading to the expression of functional CFTR protein in lung tissues. These findings underline the efficacy of the LNP design in overcoming pulmonary delivery barriers, ensuring effective mRNA transfection, and supporting the development of inhalable mRNA-based therapies. Images of the LNPs after nebulization and administration to mice, as indicated by bioluminescence signals, confirming effective transfection and protein expression, are provided in Figure 7.

#### 3.2.2. Hydrogels and Hybrid Nanoparticles

Hydrogels have also emerged as a versatile platform for drug delivery due to their unique properties, such as high water content, biocompatibility, and dynamic mechanical properties. These networks of hydrophilic polymers can swell in aqueous environments without dissolving, making them ideal carriers for a wide range of drugs, including viable and labile active drugs [50]. Hydrogels can be composed of various polymers, both natural and synthetic, with common natural polymers, including alginate, chitosan, and gelatin, while synthetic polymers include PVA, PEG, and polyacrylamide [51]. The choice of polymer affects the hydrogel’s physical properties, degradation rate, and compatibility with the encapsulated drug [52].

The stability of drugs within hydrogels is vital, especially for labile drugs. Hydrogels provide a protective environment that can shield drugs from degradation caused by environmental factors such as pH, temperature, and enzymatic activity. In a study presenting a novel method to improve siRNA delivery for respiratory disease treatment via inhalation, the research addressed the critical challenge of efficient intracellular delivery of siRNA therapies, which hold significant therapeutic potential [53]. The developed approach involved hybrid nanoparticles with a core of siRNA-loaded nanogels coated with a proteolipid shell containing surfactant protein B (SP-B). Hybrid nanoparticles are a solution that combines the benefits of both organic and inorganic materials to create multifunctional systems with enhanced therapeutic and diagnostic capabilities. This platform often utilizes a combination of polymers, such as PLGA, PCL, PEG, and lipids, to create a stable and effective drug delivery system. These polymers form the core of the nanoparticles, providing structural integrity and controlled release properties. The lipid component, typically phospholipids like hydrogenated soy phosphatidylcholine (HSPC) and DSPE-PEG2000, forms a shell around the polymeric core, enhancing biocompatibility and reducing immunogenicity [54,55].

The study systematically tested various proteolipid compositions to pinpoint components that enhance siRNA delivery, focusing on SP-B. Results indicated that SP-B significantly improves siRNA delivery and gene silencing in lung epithelial cells in vitro. Additionally, in vivo tests using a murine acute lung injury model demonstrated that SP-B enhanced siRNA delivery to alveolar macrophages and effectively reduced tumor necrosis factor-alpha levels. These findings prove that SP-B’s role is a key enhancer of siRNA delivery, thereby significantly improving the efficacy of siRNA-loaded nanogels for inhalation therapy. This study additionally addressed the critical need for efficient and safe siRNA therapies for inhalation while also emphasizing the potential of bio-inspired materials in advancing drug delivery systems for pulmonary diseases. Specifically, the nanogels were employed as the delivery vehicle for siRNA, with their surfaces coated with SP-B. This coating enhances compatibility with lung tissues, improving the stability and efficacy of the siRNA during delivery.

#### 3.2.3. Vaccine Formulations

mRNA vaccines offer a promising approach for respiratory disease immunization by encoding antigens that trigger immune responses [84]. While traditional mRNA vaccine delivery has focused on intramuscular administration, novel pulmonary delivery strategies are being developed to overcome lung-specific barriers such as mucociliary clearance, enzymatic degradation, and immune surveillance [85].

To enhance lung-targeted mRNA vaccine delivery, advanced nanocarriers have been investigated. In a study by Popowski et al. [86], pulmonary limitations are addressed. This includes the development of an inhalable mRNA vaccine system utilizing lung-derived extracellular vesicles (Lung-Exos), which demonstrated superior pulmonary retention, immune activation, and bioactive protection compared to traditional lipid nanoparticle formulations. Lung-Exos were optimized to efficiently penetrate pulmonary mucus and reach deep lung tissues. The study compared the biodistribution of Lung-Exos with conventional lipid nanoparticles (Lipos) following nebulization and dry powder inhalation (DPI) in mice and non-human primates (NHPs). Results indicated that Lung-Exos had significantly higher retention in the bronchioles and alveolar parenchyma, outperforming synthetic nanoparticles. The natural lung-derived signature of these exosomes allowed for enhanced bioavailability and reduced clearance, making them a highly efficient pulmonary delivery vehicle.

To further optimize pulmonary retention and evade mucociliary clearance, the study utilized a lyophilized dry powder formulation. Unlike conventional liquid formulations requiring cold-chain storage, lyophilized Lung-Exos remained stable at room temperature for up to 28 days, making them a more practical and scalable solution for pulmonary vaccine administration. The study also demonstrated the protective ability of Lung-Exos in preserving mRNA vaccine integrity. The SARS-CoV-2 spike (S) protein-encoding mRNA was encapsulated within Lung-Exos, forming S-Exos, which were compared against liposome-encapsulated mRNA vaccines (S-Lipo). Following inhalation, S-Exos exhibited greater antigen stability and cellular uptake in the lungs compared to S-Lipo, confirming their efficacy in protecting fragile mRNA cargo from degradation.

In murine models, S-Exos elicited higher IgG and secretory IgA (SIgA) antibody titers in the bronchoalveolar and nasopharyngeal lavage fluids compared to S-Lipo, suggesting a stronger and more durable immune response. Notably, S-Exos provided superior protection against pseudoviral challenge, demonstrating a greater ability to neutralize SARS-CoV-2 pseudoviruses. These findings indicate that lung-derived exosome vaccines offer enhanced mucosal immunity, which is crucial for preventing respiratory infections at the primary site of entry.

The findings of Popowski et al. showed the potential of extracellular vesicles as an optimized pulmonary vaccine platform. By capitalizing on mucus-penetrating properties, enhanced lung retention, and long-term stability, the Lung-Exos overcome major pulmonary barriers that limit traditional inhaled vaccines. Their ability to efficiently deliver mRNA vaccines, activate efficient immune responses, and remain stable at room temperature positions them as a promising alternative to existing mRNA vaccine formulations for respiratory diseases. Further clinical validation is, however, necessary to explore their full potential in human applications and large-scale vaccine deployment.

### 3.3. Platforms for Increased Bacterial Viability

Probiotics have emerged as a promising therapeutic strategy for respiratory health, particularly in modulating lung microbiota, enhancing immune responses, and combating respiratory infections [11,87,88,89]. However, pulmonary probiotic delivery is challenged by various pulmonary barriers, necessitating the development of advanced delivery systems to enhance stability, targeting, and therapeutic efficacy [90,91]. Recent studies have demonstrated that nanoparticle-based probiotic formulations can overcome these barriers and improve pulmonary health outcomes.

A study by Fu et al. explored a *Lactobacillus rhamnosus*-based nanoparticle system (OASCLR) for pulmonary administration, demonstrating significant therapeutic potential for bacterial pneumonia [92]. This system was designed with a mucoadhesive chitosan and hyaluronic acid coating, which facilitated prolonged retention in lung tissues by targeting inflammatory macrophages through CD44 receptor interactions. Additionally, the system was functionalized with ononin, a plant-derived antioxidant, to protect probiotics from reactive oxygen species (ROS) and oxidative stress, thereby improving their viability in inflamed lung environments. The OASCLR probiotic nanoparticle system demonstrated significant antibacterial and immune-modulating effects in an in vivo murine model of bacterial pneumonia. The formulation achieved an over 99.97% reduction in lung pathogens, confirming its potent antibacterial activity. In terms of immune modulation, the nanoparticles effectively suppressed excessive inflammation in hyperactive immune responses while enhancing macrophage phagocytosis in immune-compromised conditions, thereby promoting balanced immune function. Additionally, treatment resulted in an increase in beneficial bacteria and a reduction in pathogenic bacterial strains, restoring microbial homeostasis in the lungs. The system also facilitated macrophage reprogramming, shifting their profile from a pro-inflammatory (M1) to an anti-inflammatory (M2) phenotype, further improving immune regulation and reducing lung tissue damage associated with chronic inflammation. These outcomes highlight the potential of pulmonary probiotic therapy in managing bacterial pneumonia and improving respiratory immune resilience.

To further enhance pulmonary efficacy, the *Lactobacillus rhamnosus* core actively reduced pathogenic bacterial populations while promoting beneficial microbial diversity, which is essential for lung homeostasis. These findings align with research by Balta et al. [93], who highlighted the gut–lung axis as a critical mechanism in pulmonary probiotic therapy. The study emphasized that probiotic strains, such as *Lactobacillus* and *Bifidobacterium*, contribute to mucosal immunity by increasing secretory IgA production, thereby neutralizing respiratory pathogens. Furthermore, probiotic-derived short-chain fatty acids (SCFAs), including butyrate, have been shown to reduce airway inflammation and strengthen lung barrier integrity, displaying the systemic benefits of probiotics beyond localized lung delivery [93].

**Figure 8:**
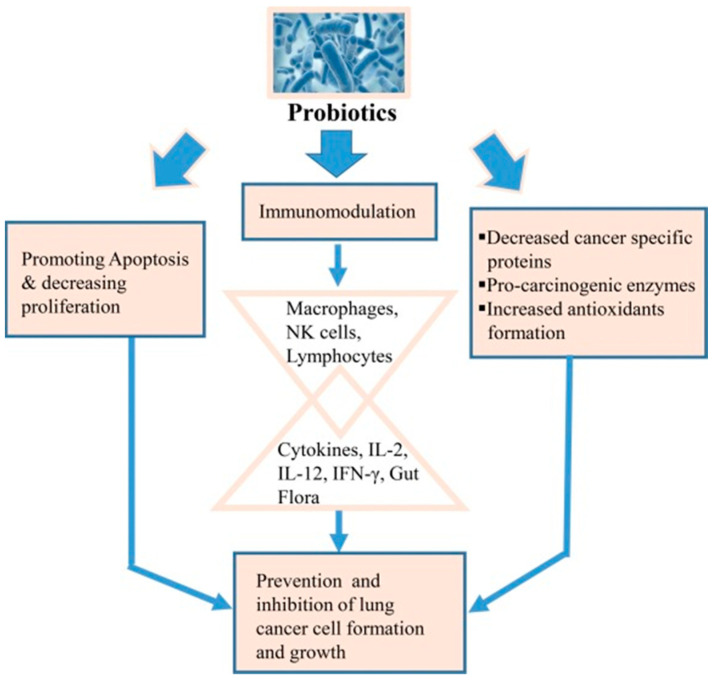
Schematic representation of how probiotics contribute to lung cancer prevention through three mechanisms (reproduced with permission from Sharma et al. [94], © 2017 Elsevier Ltd., London, UK).

Probiotics also contribute significantly to immune modulation by activating macrophages, natural killer (NK) cells, and lymphocytes, which stimulate cytokine production (IL-2, IL-12, IFN-γ) and enhance antioxidant formation, as depicted in Figure 8 [94,95]. This results in the prevention of cancer cell proliferation and the promotion of apoptosis, illustrating the broader potential of probiotics in targeting respiratory diseases, including lung cancer. Clinical studies further provide growing evidence of probiotics’ benefits in treating gastrointestinal disorders [95], respiratory infections [96], and immune system modulation [97], highlighting their potential in targeted drug delivery systems for a variety of conditions [98,99].

## 4. Clinical Studies and Marketed Products

Clinical trial analysis of pharmaceutical products is crucial in determining the efficacy and safety of potential platforms for therapeutic applications. In the context of nanosystems for the pulmonary delivery of labile bioactives, a few clinical trials, as provided in Table 3, have evaluated inhalable nanocarriers for conditions such as cystic fibrosis, diabetes mellitus type 1, and viral infections. These studies highlight the diverse translational potential of these innovations. To date, no inhalable nanosystems loaded with labile bioactives for pulmonary administration have been granted regulatory approval, however after the successes of products such as Arikayce^®^ (Insmed Inc., Bridgewater, NJ, USA; amikacin-loaded liposomes) for the treatment of lung infections caused by Mycobacterium avium complex and Curosurf^®^ (Chiesi USA Inc., Cary, NC, USA), a liposomal formulation containing surfactant proteins SP-B and SP-C for endotracheopulmonary instillation, these labile-loaded nanoplatforms have the potential to be marketed for the treatment of a variety of conditions.

## 5. Trends and Future Perspectives

Despite substantial advancements in pulmonary drug delivery, several challenges persist. While nanocarriers offer promise, inter-individual variability, including age, genetic differences, and pre-existing lung conditions, affects drug absorption and therapeutic outcomes, necessitating personalized approaches to pulmonary drug delivery. Future advancements in pulmonary drug delivery can focus on integrating biomimetic materials, such as surfactant proteins and mucus-penetrating nanoparticles, to enhance the stability and bioavailability of labile bioactives. Microfluidics and AI-driven modeling can enhance the design and optimization of nanocarriers by enabling precise control over particle size, surface charge, and drug release kinetics. These technologies facilitate the development of highly stable, biocompatible formulations that can overcome physiological barriers such as mucociliary clearance and enzymatic degradation. Furthermore, AI-driven predictive modeling allows for the customization of nanocarrier properties based on patient-specific factors, optimizing drug delivery efficiency and therapeutic outcomes for diverse patient populations.

The incorporation of multifunctional nanocarriers capable of targeted delivery, controlled release, and simultaneous imaging additionally holds potential for theragnostics, while hybrid delivery systems, such as lipid-polymer nanoparticles, can address the limitations of single-material carriers by combining their properties. The development of inhalable gene editing therapies utilizing CRISPR-Cas9 or siRNA further represents a frontier for treating genetic respiratory disorders. Collaborations between materials scientists, biologists, and clinicians will therefore be essential to translate these technologies from bench to bedside, ensuring they are not only effective but also cost-efficient and accessible.

## 6. Conclusions

The pulmonary route presents an efficient and non-invasive platform for delivering labile bioactives, yet its success is contingent upon overcoming physiological, chemical, biological, and immunological barriers that limit drug retention and bioavailability. Mucociliary clearance, enzymatic degradation, and immune responses significantly challenge the stability and efficacy of inhaled therapeutics. This review highlights the advancements in nanoparticles, liposomes, solid lipid nanoparticles, hydrogels, and hybrid nanocarriers that have been strategically designed to protect bioactives from degradation, enhance lung retention, and enable targeted and sustained drug release. Through continued advancements, pulmonary drug delivery systems will enable safer and more effective treatments, improving the management of respiratory and systemic diseases by ensuring stability, retention, and precise bioactive delivery.

## Figures and Tables

**Table 2:** Characteristics and applications of the advanced drug delivery systems for the pulmonary delivery of labile bioactives.

Delivery System	Local and/or Systemic Effects	Key Features	Benefits	Bioactives Loaded	References
Polymeric Nanoparticles	Predominantly localSystemic	Biodegradable, biocompatible, controlled release.	Protects bioactives from degradation, provides controlled, localized, and sustained release.	Probiotics, proteins, and peptides.	[39,40,41,42,43]
Solid Lipid Nanoparticles	LocalSystemic	Solid core, stabilized with surfactants.	Improved stability, prolonged release, biocompatible.	Proteins (e.g., insulin).	[44,45]
Polymeric Micelles	Predominantly localSystemic	Amphiphilic block copolymers, core–shell structure.	Improves solubility, stability, and bioavailability.	Hydrophobic drugs, mRNA.	[46,47,48,49]
Hydrogels	Local	Hydrophilic polymers, high water content.	Protects drugs, can provide controlled release, biocompatible.	Delivery of proteins (e.g., interleukin-10), peptides.	[50,51,52]
Hybrid Nanoparticles	Local	Combines polymers and lipids, multifunctional.	Enhanced stability, targeted delivery, reduced toxicity.	Delivery of chemotherapeutics (e.g., erlotinib).	[53,54,55]
Liposomes	Predominantly localSystemic	Phospholipid vesicles, biocompatible, versatile.	Enhances stability, targeted delivery, minimizes side effects.	Delivery of proteins (e.g., interleukin-2), mRNA.	[56,57,58]

**Table 3:** Clinical trials investigating inhalable nanosystem-based pulmonary drug delivery systems.

Trial ID	Type of Nanosystem	Loaded Bioactive	Condition/Disease	Phase
NCT06747858	Lipid nanoparticles	Biological: ARCT-032	Cystic fibrosis	II
ISRCTN15391202	Lipidoid nanoparticle	mRNA: ETH47	Antiviral therapy Uncontrolled asthma	I
NCT04491240	Native exosome suspension	EXO 1 inhalation EXO 2 inhalation	Coronavirus pneumonia	I/II
NCT02527265	Technosphere microparticles	Insulin	Diabetes mellitus type 1	II

## Abbreviations

The following abbreviations are used in this manuscript:

2HPβCD	2-hydroxypropyl-beta-cyclodextrin
CFTR	Cystic fibrosis transmembrane conductance regulator
COPD	Chronic obstructive pulmonary disease
DPIs	Dry powder inhalers
EPR	Enhanced permeability and retention
hGH	Human growth hormone
IFNα	Interferon-alpha
LNPs	Lipid nanoparticles
mAb	Monoclonal antibody
mRNA	Messenger RNA
NK	Natural killer
NPMPs	Nanoporous/nanoparticulate microparticles
PAP	Protein papain
PCL	Poly(caprolactone)
PEG	Polyethylene glycol
PEO	Poly(ethylene oxide)
PLGA	Poly(lactic-co-glycolic acid)
PVA	Polyvinyl alcohol
rhDNase	Recombinant human deoxy-ribonuclease
siRNA	Small interfering RNA
SLNs	Solid lipid nanoparticles
SP-B	Surfactant protein B
SSL	Sterically stabilized liposomes
